# How to Recognize and Treat Neoplastic Meningitis

**Published:** 2013-05-01

**Authors:** Margaret M. Fields

**Affiliations:** From MD Anderson Cancer Center, Houston, Texas

As length of survival time for many cancers has increased through the years, there has been a subsequent increase in uncommon metastatic sites. One of these sites is leptomeningeal metastases, which is cancer that has spread to the meninges, or covering of the brain, and the surrounding cerebrospinal fluid (CSF). Metastasis in this location are known by several names (Walz, 2011): neoplastic meningitis (NM), leptomeningeal disease (LMD), leptomeningeal carcinomatosis (LC), carcinomatous meningitis (CM), or meningeal carcinomatosis (MC).

This metastatic site was first described in 1870 in a patient with lung cancer (Kesari & Batchelor, 2003). It now affects between 5% and 15% of cancer patients. Most commonly affected are those with solid tumor malignancies of the breast and lung, melanoma, as well as hematologic malignancies, mainly acute lymphocytic leukemia (Lombardi et al., 2011; Jiménez Mateos et al., 2011; Meriggi & Zaniboni, 2011; Walz, 2011). Patients with primary tumors not previously likely to develop NM are now developing this condition. These include gastric, prostate, ovarian, cervical, and endometrial cancers (Mammoser & Groves, 2010).

Dissemination of malignant cells to the meninges and surrounding CSF can occur through various mechanisms. These include (1) hematogenous spread, (2) direct invasion from dural, bony, or parenchymal brain metastases, (3) cancer cells traveling along nerve pathways to reach the subarachnoid space, which occurs most often in head and neck malignancies, and (4) iatrogenesis from a surgical procedure to remove metastatic brain metastases (Mammoser & Groves, 2010; Omar & Mason, 2008).

## Diagnosis

**CLINICAL SIGNS AND SYMPTOMS**

It is important for clinicians to maintain an index of suspicion for this condition, as it is often underdiagnosed. Autopsy has shown NM to be present in up to 19% to 25% of cases of malignant disease (Strik & Prommel, 2010; Omar & Mason, 2008). Parenchymal central nervous system metastases and bony metastases are present in up to 50% of patients with NM. Clinicians may overlook the development of NM in a patient with known metastases in these locations and mistakenly attribute new symptoms to these other disease sites (Omar & Mason, 2008). Neoplastic meningitis may present in a variety of ways. It may be an asymptomatic finding upon imaging, a single neurologic deficit such as ataxia or diplopia, or multifocal neurologic deficits. It can also present as radicular signs of weakness and paresthesia.

Neoplastic meningitis is diagnosed based on three factors: (1) clinical symptoms, (2) radiologic imaging (typically MRI), and (3) analysis of the CSF. Symptoms will vary based on the location of lesions. Symptoms are often multifocal, involving cerebral, spinal, and cranial nerve components of the neuroaxis (see Table). Cerebral symptoms may include headache, confusion, behavioral changes, ataxia, incoordination, or nausea and vomiting. Spinal symptoms include muscle weakness, paresthesias, back/neck pain, or bowel and bladder dysfunction. Cranial nerve symptoms include diplopia, visual loss, facial numbness, facial weakness, tinnitus, hearing loss, hoarseness, or difficulty in swallowing. Patients may have few or no symptoms, or they may have several cranial nerve palsies—the most common being III, IV, VI, and VII—that result in diplopia, visual field loss, inability to perform full extraocular movements, or facial droop (Mammoser & Groves, 2010; Siddiqui, Marr, & Weissman, 2009; Lombardi et al., 2011).

**Table 1:**
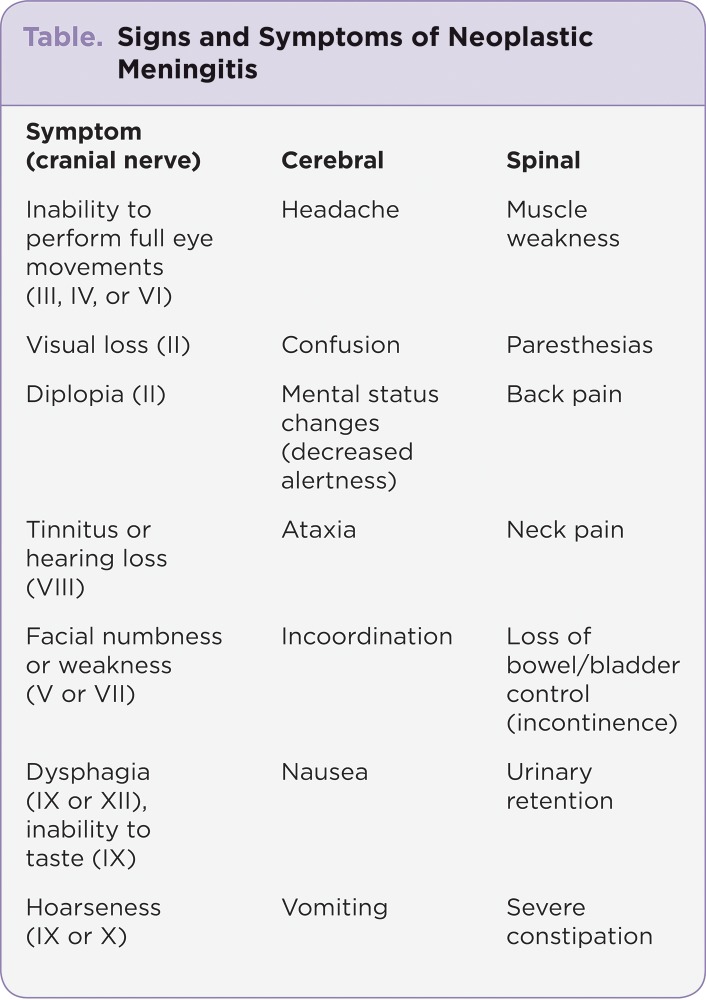
Table 1. Signs and Symptoms of Neoplastic Meningitis

**RADIOLOGIC IMAGING**

**Figure 1:**
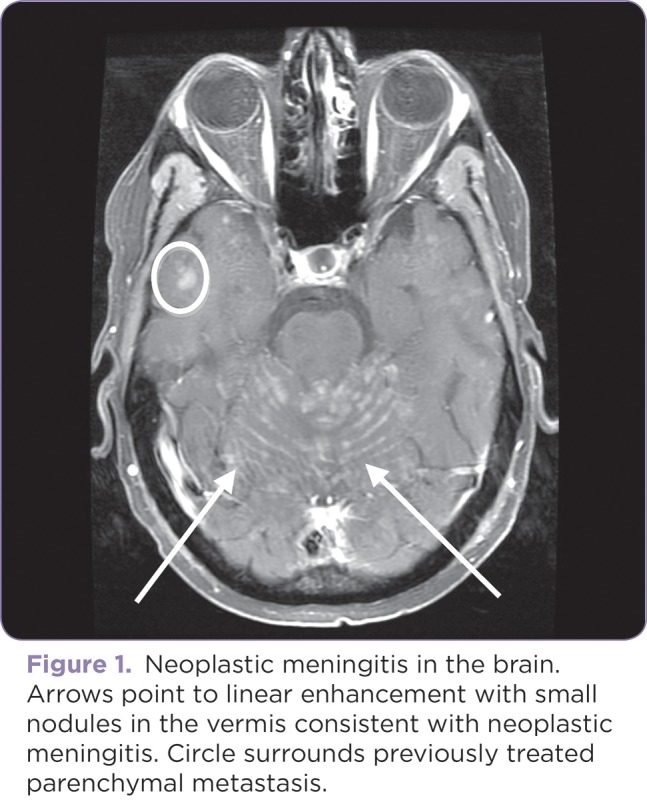
Figure 1. Neoplastic Meningitis in the brain. Arrows point to linear enhancement with small nodules in the vermis consistent with neoplastic meningitis. Circle surrounds previously treated parenchymal metastasis.

**Figure 2:**
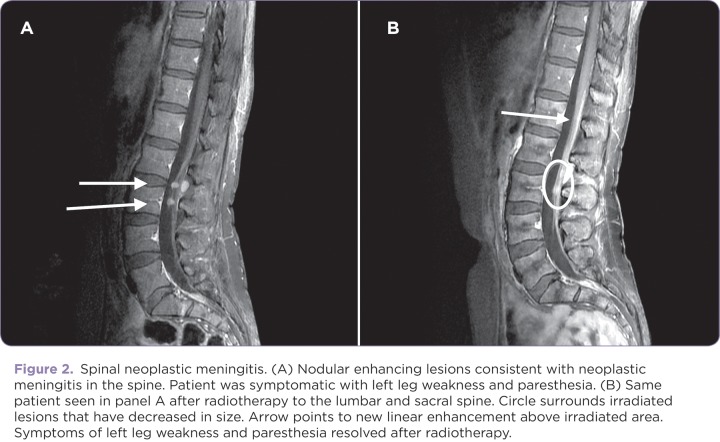
Figure 2. Spinal neoplastic meningitis. (A) Nodular enhancing lesions consistent with neoplastic meningitis in the spine. Patient was symptomatic with left leg weakness and paresthesa. (B) Same patient seen in panel A after radiotherapy to the lumbar and sacral spine. Circle surrounds irradiated lesions that have decreased in size. Arrow points to new linear enhancement above irradiated area. Symptoms of left leg weakness and paresthesia resolved after radiotherapy.

In early stages, MRI findings of NM may be difficult to detect. Typical findings are enhancement and/or enlargement of cranial nerves, nodular or linear leptomeningeal enhancement extending into sulci or basal cisterns, dural thickening, and intradural enhancing nodules along the spinal cord, especially in the cauda equina (Chamberlain, Glantz, Groves, & Wilson, 2009; Pauls et al., 2012). Contrast-enhanced T1 sequences are traditionally the series on MRI used to evaluate for meningeal enhancement and nodularity. The sensitivity and specificity of detecting NM on this MRI sequence are 59% and 93%, respectively. More recently, T1 postcontrast fluid-attenuated inversion recovery (FLAIR) sequences have been advocated for their ability to detect superficial meningeal disease; however, the sensitivity and specificity of this sequence are only 41% and 88%, respectively (Omar & Mason, 2008). Other imaging modalities that have been evaluated for diagnostic evaluation of NM include CT scan and PET-CT; neither has been shown to be equal to MRI (Lombardi et al., 2011); see Figures 1 and 2.

**CYTOLOGY**

The gold standard for diagnosis of NM is positive cytology. However, due to the low sensitivity (50%) associated with a single lumbar puncture (LP), there may be no malignant cells detected in a sample even when the patient has NM. The sensitivity of cytology from LP rises to 90% when three lumbar punctures are performed. The likelihood of obtaining positive cytology also increases if the LP is performed close to the area of abnormality on imaging and the CSF is sent to the laboratory on ice to be evaluated as soon as possible. This process increases accuracy, as the lysis of cells begins within 1 hour. It is important to send an ample amount of fluid for analysis—a minimum of 10.5 mL—to increase the sensitivity as well (Deisenhammer et al., 2006; Mammoser & Groves, 2010).

It can be difficult to discern between malignant cells and inflammatory cells, so immunohistochemistry staining techniques are sometimes used to obtain a definitive diagnosis. Specific tumor markers that can be used to confirm a diagnosis of NM are carcinoembryonic antigen (CEA) for carcinoma; human chorionic gonadotropin (beta-hCG) for choriocarcinoma, embryonal carcinoma, or germ cell tumor; alpha-fetoprotein (AFP) for teratocarcinoma, yolk sac tumor, embryonal carcinoma, or endodermal sinus tumor; CA-125 for ovarian cancer; CA 15-3 for breast cancer; prostate-specific antigen (PSA) for prostate cancer; and melanin for melanoma. Nonspecific markers that are elevated in the presence of malignancy, but not specific to a certain cancer, are beta-microglobulin, beta-glucuronidase, or lactate dehydrogenase (Omar & Mason, 2008). If the cytology is negative in the presence of clear imaging findings and clinical symptoms, the diagnosis of NM may be made based on the combination of imaging findings and clinical symptoms alone (Chamberlain et al., 2009; Mammoser & Groves, 2010; Walbert & Groves, 2010). In instances of negative cytology in the presence of convincing radiologic or symptomatic findings, it is hypothesized that the tumor cells are adherent to the membranes and infrequently exfoliate into the surrounding fluid (Omar & Mason, 2008). Serial cytologic analysis during treatment can be used to assess response to therapy and to detect relapse (Omar & Mason, 2008).

## Treatment

Overall, NM carries a very poor prognosis. If untreated, survival is 4 to 6 weeks; with aggressive therapy, survival is 2 to 6 months. Despite this, there are a few long-term survivors (Mammoser & Groves, 2010). Therapy is palliative, with the goal of preventing further neurologic decline and extending survival. It is unlikely to reverse any fixed neurologic deficits such as radicular weakness or cranial nerve palsies (Mammoser & Groves, 2010; Omar & Mason, 2008). A key factor in outcome is early diagnosis, as NM responds to treatment better in the early phase of development (DeAngelis & Boutros, 2005).

Several retrospective reviews have been conducted to determine prognostic factors to better predict, at the time of diagnosis, who is likely to benefit from therapy. Negative prognostic factors are older age (> 65); poor performance status (Karnofsky performance status ? 70); bulky CNS disease; persistent CSF flow abnormalities; extensive systemic disease with limited treatment options; multiple, severe, fixed neurologic deficits; and encephalopathy (Chamberlain, 2008; de Azevedo et al., 2011; Gauthier et al., 2010; Mammoser & Groves, 2010; Oechsle, Lange-Brock, Kruell, Bokemeyer, & de Wit, 2010). Patients with NM and a primary malignancy of leukemia or lymphoma have the best overall prognosis and should be aggressively treated. Patients with breast cancer appear to have the best response among the solid tumors, with up to 25% of patients achieving 1-year survival. Patients with small cell lung cancer have also been shown to attain a positive response from therapy. Patients with the worst outcomes are those with non–small cell lung cancer and melanoma (Omar & Mason, 2008).

Treatment for NM comprises any or all of the following three components: (1) radiotherapy, (2) intraventricular/intrathecal chemotherapy, and (3) systemic chemotherapy (Demopoulos & Brown, 2011; Meriggi & Zaniboni, 2011; Strik & Prommel, 2010). Oechsle et al. (2010) have shown a survival benefit for patients receiving intraventricular chemotherapy combined with systemic therapy (median survival, 5.6 months) and for patients receiving all three therapies (median survival, 5.8 months) when compared to intraventricular chemotherapy alone (median survival, 1.4 months) or to intraventricular chemotherapy combined with radiotherapy (median survival, 2 months). This highlights the importance of added systemic therapy.

**RADIOTHERAPY**

Radiotherapy is directed at sites of bulky disease or symptoms such as pain, paresthesias, or cranial nerve palsies. Since NM involves the entire neuroaxis, complete craniospinal radiotherapy would be required to treat all potential areas of involvement. This is generally not performed, as it carries a high morbidity and may cause profound myelosuppression, resulting in an inability to administer systemic chemotherapy at a later date (Omar & Mason, 2008). Radiotherapy may be given intermittently throughout the course of therapy as new symptoms or imaging findings emerge. Radiotherapy is generally given in short courses of 20 to 30 Gy in 5 to 10 fractions (Demopoulos & Brown, 2011; Strik & Prommel, 2010).

**INTRATHECAL/INTRAVENTRICULAR CHEMOTHERAPY**

Despite a lack of convincing evidence, intraventricular chemotherapy via an Ommaya reservoir or intrathecal chemotherapy via lumbar puncture is the mainstay of treatment. Administration directly into the CSF allows chemotherapy to circulate throughout the entire neuroaxis and avoids the issue of getting chemotherapy across the blood-brain barrier. Generally, an Ommaya reservoir is placed at the beginning of therapy to facilitate ease of administration due to the need for frequent and ongoing treatments. Following placement of the Ommaya reservoir and prior to initiation of chemotherapy, a CSF flow study is conducted with injection of a radioactive tracer to assess for any areas of blockage. If blockage is identified, it should be treated with radiotherapy to relieve the obstruction and improve CSF flow. Similarly, areas of bulky or nodular disease require radiotherapy, as chemotherapy given into the CSF penetrates only 2 to 3 mm into the tissue and nodular disease will be inadequately treated (Mammoser & Groves, 2010; Strik & Prommel, 2010).

Various drugs have been studied for use as intraventricular/intrathecal chemotherapy. These include chemotherapy agents such as cytarabine, liposomal cytarabine (Depocyt), methotrexate, topotecan, thiotepa, etoposide, gemcitabine, busulfan, and immunotherapy agents such as alpha-interferon and interleukin-2. More recently, targeted monoclonal antibodies (rituximab [Rituxan] and trastuzumab [Herceptin]) have been evaluated as well.

Side effects of therapy vary but are generally local in nature, with chemical meningitis being the most common. Symptoms of chemical meningitis include headache, fever, and nausea (Chamberlain, 2008; Lombardi et al., 2011). Therapy is generally initiated two times per week, with the exception of liposomal cytarabine, which is given every 2 weeks. Following a 6- to 12-week induction period, treatment frequency is decreased to weekly for an additional 6 to 12 weeks of consolidation, and then further decreased to monthly maintenance therapy if the patient is tolerating treatment well and the disease is stable (Chamberlain, 2008).

At present, the most commonly used intraventricular/intrathecal chemotherapies are methotrexate, cytarabine, liposomal cytarabine, and topotecan. Gemcitabine is no longer used for intraventricular/intrathecal therapy due to significant neurotoxicity causing somnolence (Lombardi et al., 2011). Glantz, Van Horn, Fisher, and Chamberlain (2010) noted a difference in progression-free survival (PFS) related to route of administration (intrathecal vs. intraventricular) with short half-life chemotherapy such as methotrexate. In the methotrexate group, PFS was 19 days in the intrathecal group and 43 days in the intraventricular group. The hypothesis is that the transit of short half-life chemotherapy drugs is too brief to allow for adequate back-diffusion into the ventricles where malignant cells are known to reside (Chamberlain, Kormanik, & Glantz, 2001; Glantz et al., 1998). There have been no other studies that compare route of administration and PFS; this topic warrants additional study to optimize therapy and outcomes in this patient population.

More recently, promising results have been seen in monoclonal antibody therapy with rituximab in lymphoma, and with trastuzumab in breast cancer, glioblastoma, and medulloblastoma (Perissinotti & Reeves, 2010). In a phase I study, Rubenstein et al. (2007) demonstrated activity in 10/10 patients with CNS lymphoma who received intraventricular rituximab. Six patients had cytologic response, and four patients had complete response. Schulz et al. (2004) reported on the use of intraventricular rituximab in six patients with relapsed CNS lymphoma, four of whom had meningeal involvement. He reported total clearing of meningeal tumor cells in three of the four patients with meningeal involvement and a complete remission in the fourth patient. There was a minor response in one of the patients with cerebral metastasis and parenchymal progression in the remaining patient who had primary CNS lymphoma.

Allison and colleagues (2009) treated 16 patients with NM from breast cancer (4), glioblastoma (11), or medulloblastoma (1) with a 62.5% cytologic, radiologic, or clinical response. Two of four breast cancer patients, seven of eleven glioblastoma patients, and the single medulloblastoma patient were responders. In a review article, Perissinotti and Reeves (2010) noted survival durations of 39 days to greater than 72 months in patients receiving intrathecal trastuzumab and survival durations of 1.1 weeks to greater than 3.5 years in patients receiving intrathecal rituximab. These therapies continue to be evaluated in the clinical trial setting and are not standard treatment at this time.

**SYSTEMIC CHEMOTHERAPY**

Concomitant systemic chemotherapy is indicated for patients who have evidence of systemic disease. Certain drugs, such as topotecan, capecitabine, temozolomide, and gemcitabine, along with high-dose methotrexate or cytarabine, have the ability to cross the blood-brain barrier and can provide dual benefit. Ultimately, systemic therapy should be any drug or combination of drugs likely to have benefit in the primary malignancy (Omar & Mason, 2008). There is no significant myelosuppression from intraventricular or intrathecal chemotherapy, so this does not need to be a consideration in selection of systemic therapy. Treatment with systemic therapy can be challenging in these patients as they have often been heavily pretreated and may have limited bone marrow reserve. Systemic therapy is a key component of treatment, as 22% to 25% of patients die from progression of both NM and their systemic disease and 19% to 44% of patients eventually die as a result of their systemic disease (Glantz et al., 2010; Mammoser & Groves, 2010; Oechsle et al., 2010).

## Conclusion

Neoplastic meningitis needs to remain in the differential diagnosis of any cancer patient with new, particularly multifocal, neurologic symptoms. While this diagnosis carries a poor prognosis, early diagnosis and treatment can prevent neurologic deficits and extend survival with good quality of life.
